# Stochastic mutation as a mechanism for the emergence of SARS-CoV-2 new variants

**DOI:** 10.1016/j.virusres.2025.199667

**Published:** 2025-11-20

**Authors:** Liaofu Luo, Jun Lv

**Affiliations:** a Faculty of Physical Science and Technology, Inner Mongolia University, Hohhot 010021, China; b College of Science, Inner Mongolia University of Technology, Hohhot 010051, China

**Keywords:** SARS-CoV-2, Spike protein mutation, Evolutionary analysis

## Abstract

Predicting the future evolutionary trajectory of SARS-CoV-2 remains a critical challenge, particularly due to the pivotal role of spike protein mutations. It is therefore essential to develop evolutionary models capable of continuously integrating new experimental data. In this study, we employ a cladogram algorithm that incorporates established assumptions for mutant representation — using both four-letter and two-letter formats — along with an *n*-mer distance algorithm to construct a cladogenetic tree of SARS-CoV-2 mutations. This tree accurately captures the observed changes across macro-lineages. We introduce a stochastic method for generating new strains on this tree based on spike protein mutations. For a given set *A* of existing mutation sites, we define a set *X* comprising *x* randomly generated mutation sites on the spike protein. The intersection of *A* and *X*, denoted as set *Y*, contains *y* sites. Our analysis indicates that the position of a generated strain on the tree is primarily determined by *x*. Through large-scale stochastic sampling, we predict the emergence of new macro-lineages. As *x* increases, the dominance among macro-lineages shifts: lineage O surpasses N, P surpasses O, and eventually Q surpasses P. We identify threshold values of *x* that delineate transitions between these macro-lineages. Furthermore, we propose an algorithm for predicting the timeline of macro-lineage emergence. In conclusion, our findings demonstrate that SARS-CoV-2 evolution adheres to statistical principles: the emergence of new strains can be driven by randomly generated spike protein sites, and large-scale stochastic sampling reveals evolutionary patterns underlying the rise of distinct macro-lineages.

## Highlights

• Constructed a cladogram elucidating evolutionary relationships among mutants.
• Deduced principles governing the emergence of novel macro-lineages.
• Demonstrated stochastic mutation drives new SARS-CoV-2 variants emergence.

## 1 Introduction

Over the past five years, Severe Acute Respiratory Syndrome Coronavirus 2 (SARS-CoV-2) has undergone continuous mutation, giving rise to numerous variants such as Alpha, Beta, Gamma, Delta, and Omicron. The complex interplay among viral antigenicity, transmissibility, and virulence complicates predictions regarding the future trajectory and disease burden of Coronavirus Disease 2019 (COVID-19) (Carabelli et al., 2023; Markov et al., 2023; Cao et al., 2023; Mallapaty, 2022; Callaway, 2021; Wang et al., 2023). Recent studies have introduced innovative approaches for forecasting pandemic evolution. For example, a network-based inference method has been proposed for short- to medium-term predictions, though its reliability decreases for long-term forecasting (Rahnsch and Taghizadeh, 2024). Despite the substantial contribution of non-spike genomic regions (e.g., ORFla, ORFlb, N, and M genes) and host immune pressures to lineage differentiation, non-synonymous mutations in the spike protein represent the largest proportion of evolutionary mutations in viruses. Furthermore, the spike protein also plays a crucial role in viral transmissibility and immune escape. Given the crucial role of spike protein mutations in SARS-CoV-2 evolution, deep learning techniques—particularly those utilizing Large Language Models—have been developed to predict future protein sequences (Ramachandran et al., 2024; Fowler and Fields, 2014; Elnaggar et al., 2022; Ferruz et al., 2022). Among these, the PandoGen algorithm has shown promise in training protein language models for pandemic-related protein forecasting (Ramachandran et al., 2024). However, challenges persist in applying PandoGen to predict recombinant SARS-CoV-2 lineages and in ensuring the continuous incorporation of new experimental data.

Recent literature has emphasized the influence of biological driving factors on mutational probability (Ma et al., 2023; Sultana et al., 2024; Yao et al., 2024; Ma et al., 2024; Guo et al., 2025). Viral evolution is primarily driven by two key factors: intrinsic transmissibility, determined by SARS-CoV-2′s angiotensin-converting enzyme 2 (ACE2) binding affinity, and immune evasion. Studies have observed numerous immune-evasive mutations in Omicron subvariants, indicating that immune pressure correlates with the accumulation of immune escape mutations, with similar pressure patterns observed among variants within the same macro-lineage. Additionally, computational analyses of affinity dynamics have been explored. These findings collectively contribute to the refinement of our model.

Building upon the driving force of immune evasion and its relationship with affinity dynamics, we propose an evolutionary model for viral mutations. First, utilizing the 4-letter mutant representation and its relationship with the 2-letter representation, along with a robust tree construction algorithm, we construct an evolutionary tree that accurately reflects observed evolutionary patterns of existing viral strains. Subsequently, integrating the concept of stochastic immune escape mutation, we develop a statistical method termed the A-X model (Luo and Lv, 2024), extending the utility of the evolutionary tree to predict the emergence of novel macro-lineages.

Our findings indicate that the probability of macro-lineage emergence correlates with the number of stochastically mutated sites on the spike protein. As this number increases, macro-lineage proportions shift sequentially: lineage O surpasses N, followed by P surpassing O, and ultimately Q surpassing P. We successfully predicted the emergence of macro-lineage P, which has since been validated.

In a previous study (Luo and Lv, 2023), we developed a mathematical model to analyze COVID-19 transmission dynamics, particularly focusing on explaining the second wave of SARS-CoV-2 infections and investigating competitive transmission between two viral strains within a region. Crucially, predicting new macro-lineage emergence is closely linked to such competitive dynamics. These results provide a vital theoretical foundation for understanding SARS-CoV-2 evolution.

A further challenge involves accurately predicting the evolutionary timeline of SARS-CoV-2. To forecast the future trajectory of macro-lineage P and the potential emergence of a macro-lineage Q, it is essential to understand how the number of mutated sites on the spike protein of selected SARS-CoV-2 variants changes over time. Despite the complexity of factors influencing viral evolution, we observed an approximately linear relationship between the number of mutated sites in a given variant and the date of its first global sample collection. This relationship enables the development of an algorithm to predict the timeline of macro-lineage transformations.

## 2 Methods

### 2.1 ACE2 binding affinity of single mutations and four-letter representation of mutants

We obtained data on single-point mutations affecting the interaction between the receptor-binding domain (RBD) – spanning amino acid residues 331 to 531 of the spike protein – and the ACE2 receptor from references (Gangavarapu et al., 2023; Starr et al., 2020). For each mutation at residue i, the mean and standard deviation of the binding affinity are denoted as *b_i_* and *s_i_*, respectively. A mutation is classified as affinity-enhancing if its measured affinity *m_i_* satisfies *m_i_*>*b_i_*+*s_i_*; affinity-weakening if *m_i_*<*b_i_*−*s_i_*; and negligible in effect if *b_i_*−*s_i_*≤*m_i_*≤*b_i_*+*s_i_*.

The surface of coronaviruses is decorated with a spike protein, which in SARS-CoV-2 consists of approximately 1273 amino acids. Each SARS-CoV-2 strain is represented by a four-letter sequence, where each letter indicates the mutation type at a specific residue. The encoding is defined as follows: 0 denotes no mutation relative to the wild type; 1 indicates a mutation with negligible effect on ACE2 binding affinity; 2 represents an affinity-enhancing mutation; and 3 signifies an affinity-weakening mutation. Thus, the full spike protein of any SARS-CoV-2 mutant is represented by a 1273-character sequence using this four-letter alphabet. Note that for regions outside the RBD, only the symbols 0 and 1 are used.

### 2.2 Cladogram algorithm on the construction of the cladogenetic tree

In the four-letter representation of mutants, let *p_a_* denote the probability of a letter "*a*" (where *a* ∈ {0,1,2,3}) occurring in a sequence, and let *p_ab_* represent the joint probability of consecutive occurrence of letters "*a*" and "*b*". More generally, for any *n*-letter segment *σ* =*abc*…, let *p_σ_* denote the joint probability of the bases in *σ* occurring in the sequence. For joint probability calculations, all sequences are assumed to be circular. For any given *n*, the sum of joint probabilities over all segments *σ* of length *n* equals 1:

$$\sum_{\sigma }p_{\sigma }=1,$$

where the summation extends over all 4*^n^* possible segments of length *n*.

For two sequences Σ and Σ′ with corresponding joint probability sets {*p_σ_*} and {*p'_σ_*}, we define the *n*-mer distance between them based on joint probability differences as:

(1) $$E_{n}\left(\Sigma ,\Sigma^{\prime }\right)=\sum_{\sigma }\left|p_{\sigma }-p_{\sigma }^{\prime }\right|.\;n=1,2,\ldots$$

The arguments of *E_n_* may be omitted when unambiguous. This *n*-mer distance is well-defined for unaligned sequences of unequal lengths. By iteratively applying the relation:

(2) $$\left|p_{\sigma }-p_{\sigma }^{\prime }\right|=\left|\sum_{a}\left(p_{\sigma a}-p_{\sigma a}^{\prime }\right)\right|\leq \sum_{a}\left|p_{\sigma a}-p_{\sigma a}^{\prime }\right|,$$

where *σ* is any *n*-letter segment and *σa* denotes an (*n* + 1)-letter segment, it follows that:

(3) $$E_{n+1}\geq E_{n},\;n=1,2,\ldots$$

Three primary algorithms are commonly employed for phylogenetic tree inference: distance matrix methods, maximum parsimony analysis, and maximum likelihood estimation (Li, 1997). In this study, we utilized these approaches (primarily the unweighted pair group method with arithmetic mean, UPGMA (Nei and Kumar 2000)) to reconstruct cladogenetic relationships.

## 3 Results and discussion

### 3.1 Reconstruction of the cladogram of virus mutations

**Fig. 1:**
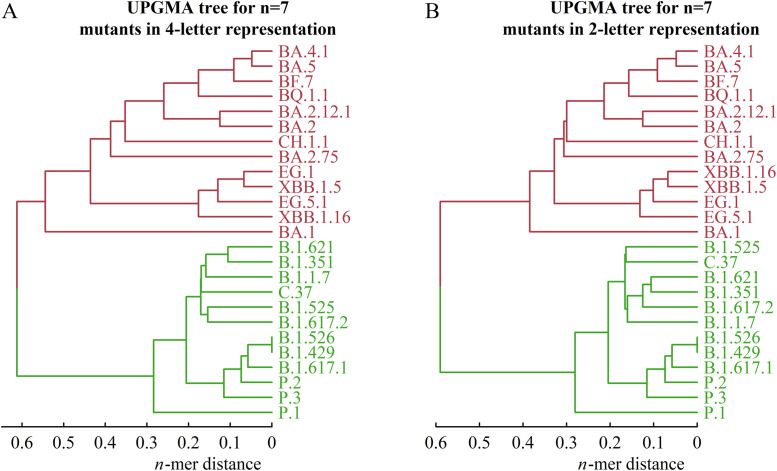
Cladogenetic tree describing virus mutation. (A) The mutants in 4-letter representation. (B) The mutants in 2-letter representation (2-letter representation is the approximation of 4-letter representation and the related explanation can be found in the following text.).

Without loss of generality, we constructed a distance matrix *D* for the 25 SARS-CoV-2 variants listed in Supplementary Material 1, Table S1 using the *n*-mer distance method. In this matrix, mutants are labeled as *i, j, k*, …, and each element *D_ij_* represents the *n*-mer distance between sequences Σ*_i_* and Σ*_j_*, where Σ*_i_* denotes the sequence of mutant *i*. By applying the UPGMA algorithm to this matrix, we reconstructed an evolutionary tree of the 25 mutants for a specific *n*. We observed that the cladogram structure stabilizes after several iterations as *n* increases, reaching a stable state when n ≥ 7 (Luo and Lv, 2024). Detailed comparisons of tree topologies for different *n* values are provided in Supplementary Material 2, Figures S1 and S2. Fig. 1 displays the cladogram for n = 7, illustrating viral mutations in both four-letter and two-letter representations.

In Fig. 1, two major branches correspond to the macro-lineages N and O of the virus strains. Within macro-lineage O, sub-branches clearly show the divergence of BA.1 into BA.2, BA.4, and BA.5. The bifurcation of BA.1 from other O-lineage strains is consistent with recent phylogenetic analyses from Nextstrain (Hadfield et al., 2018). Additionally, XBB is accurately classified as a recombinant of two BA.2 strains. In macro-lineage N, the sub-branch encompasses four variants of concern (VOCs): B.1.1.7 (Alpha), B.1.351 (Beta), B.1.617.2 (Delta), and P.1 (Gamma), each representing independent sub-lineages. These findings confirm that the theoretical tree aligns with established evolutionary characteristics of the virus strains (Wang et al., 2023).

The tree reconstruction relies on two key assumptions: the four-letter representation of mutants and the *n*-mer distance algorithm. The four-letter scheme uses binary values (0 and 1) for 104 mutational sites on the spike protein, with two additional options (2 and 3) permitted at 27 sites in the receptor-binding domain (RBD). To simplify computations, we approximated this representation using a two-letter version, ignoring options 2 and 3. A comparison between the two-letter and four-letter representations for the 25 mutants is presented in Fig. 1. Further structural comparisons of the cladograms are available in Supplementary Material 2.

Why does the two-letter representation approximate the four-letter version effectively? Due to interactions between the RBD and the human ACE-2 receptor, the 201 amino acid residues in the RBD cannot vary independently (Sultana et al., 2024). This constraint significantly reduces the informational complexity of the RBD. Thus, the two-letter approximation—which omits options 2 and 3 in the RBD—provides a reasonable simplification.

### 3.2 Generation of new strains on the cladogenetic tree using a stochastic method

Once the cladogenetic tree is constructed from known viral mutant lineages, new strains can be generated and analyzed within this framework. This section introduces a stochastic modeling approach for this process. Let set *A* comprise all mutated sites in updated virus strains, where *a* denotes the number of such sites. Each strain is represented as a two-letter sequence derived from *A*(*a*).

To model stochastic mutations on the spike protein, let *X* denote a set of *x* sites subject to stochastic sampling. The union of sets *A*(*a*) and *X*(*x*) is defined as Z = *A* ∪ X, while their intersection, containing *y* sites, is denoted *Y*(*y*). Assuming that new strains arise from stochastic sampling within *Z*, each new strain is represented by a two-letter sequence from *Z*(*len*), where *len* = a + x − y. Using a cladogram algorithm, we reconstruct the cladogenetic tree of mutants in set *Z*, which incorporates the predicted new strain. This methodology is termed the A–X model (Luo and Lv, 2024).

Importantly, the model can consistently predict new strains, regardless of how sets *A* and *X* are distributed along the sequence or how set *Y* is incorporated. It ensures accurate predictions even with the continuous addition of new experimental data. Note: Both set *X* and set *A* may encompass residue pairs, enabling the consideration of residue interactions in the A-X model (Luo and Lv, 2024).

To demonstrate the feasibility of the A-X model in predicting new SARS-CoV-2 strains, we consider the example of 25 mutants listed in Supplementary Material 1, Table S1. Assume the evolutionary tree of these 25 mutants (set *A*) has two main branches, N and O. By performing stochastic sampling *S* times (e.g., S = 10^5^), we generate 10^5^ predictions for new strains. The following observations are made:

1. The new strain may belong to either branch N or O, or lie outside both, suggesting the emergence of a new macro-lineage.
2. For a fixed *x*, different values of *y* can lead to distinct predictions regarding the position of the new strain on the tree. The position is determined by the pair (*x, y*). Thus, for a given (*x, y*), the predicted new strain is denoted as New*_x.y_*.
3. When *x* is small, the predicted new strain typically falls within branch N or O. However, once *x* exceeds a certain threshold, an anomaly occurs, indicating the emergence of a new macro-lineage to which the new strain belongs.

### 3.3 Expanding the scope of stochastic sampling and predicting new SARS-CoV-2 macro-lineages

The emergence of new strains in phylogenetic trees is a stochastic process. As the scale of stochastic sampling expands, discernible statistical patterns begin to emerge. To broaden this sampling scope, we increased the size of *x* and randomized parameter *y* within the intersection set. This approach effectively enhances stochastic information, thereby facilitating new insights into the emergence of macro-lineages.

#### 3.3.1 Generation of multiple macro-lineages

At a given time *t*, the emergence of new variants from set *A*(*a*) can be predicted. In the A–X model, set *X* is stochastically selected, resulting in the random appearance of new variants on the phylogenetic tree. Nevertheless, the generation of multiple macro-lineages and the consequent bifurcation of the tree topology into several major branches are inevitable.

**Fig. 2:**
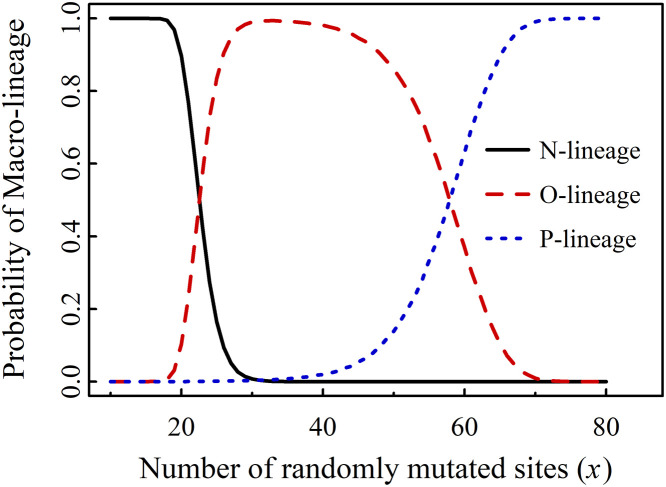
PML(Probability of Macro-lineage) vs *x* for 25 mutants (a = 104).

For example, at t = *T*_1_ (October 15, 2023; accession date of 25 mutants; see Table S1 in Supplementary Material 1), we performed 10^5^ rounds of stochastic sampling for *x* and 6 rounds of randomization for *y*. Throughout this process, the consistent emergence of macro-lineages N, O, and P was observed. The classification of a variant into macro-lineages N, O, or P is retrieved from Outbreak Info (Gangavarapu et al., 2023), based on PANGO lineage designations. The probability of a lineage (N, O, or P) for a given *x* is calculated as the occurrence count of that lineage divided by the total number of variants (10^5^). Specifically, the macro-lineage affiliation of a newly generated variant is determined by its position within the bifurcated phylogenetic tree. The results are shown in Fig. 2, which plots the probability of the *j*-lineage (j = *N*, O, P) against *x*.

A 99th percentile threshold was used in the calculation. We found that the macro-lineage assignment of a new variant depends statistically on x, with specific threshold values distinguishing different macro-lineages as follows:

New*_x.y_* ϵ N when *x* ≤ 18; New*_x.y_* ϵ N or O when 18<x ≤ 29; New*_x.y_* ϵ O when 29<x ≤ 36; New*_x.y_* ϵ O or P when 36<x ≤ 69; and New*_x.y_* ϵ P when *x* > 69.

**Fig. 3:**
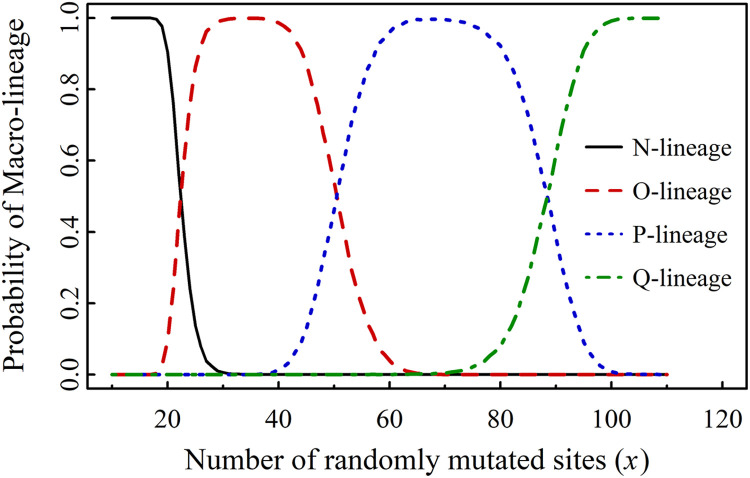
PML(Probability of Macro-lineage) vs *x* for 36 mutants (a = 128).

At a later time point, *T*_2_ (July 20, 2024), after incorporating 11 new mutants (listed in Table S2 of Supplementary Material 1) into set *A*, the value of a increased from 104 to 128, covering 36 mutants in total. We repeated the stochastic sampling process with 10^5^ iterations for *x* and 6 for *y*. The probabilities of each *j*-lineage (j = *N*, O, P, Q) are presented in Fig. 3.

Using the same method, we obtained the following threshold values of *x* for the case of 36 mutants:

New*_x.y_* ϵ N when *x* ≤ 18; New*_x.y_* ϵ N or O when 18<*x* ≤ 29; New*_x.y_* ϵ O when 29<*x* ≤ 39; New*_x.y_* ϵ O or P when 39<x ≤ 62; New*_x.y_* ϵ P when 62<x ≤ 72; New*_x.y_* ϵ P or Q when 72<x ≤ 99 and New*_x.y_* ϵ Q when *x* > 99.

**Fig. 4:**
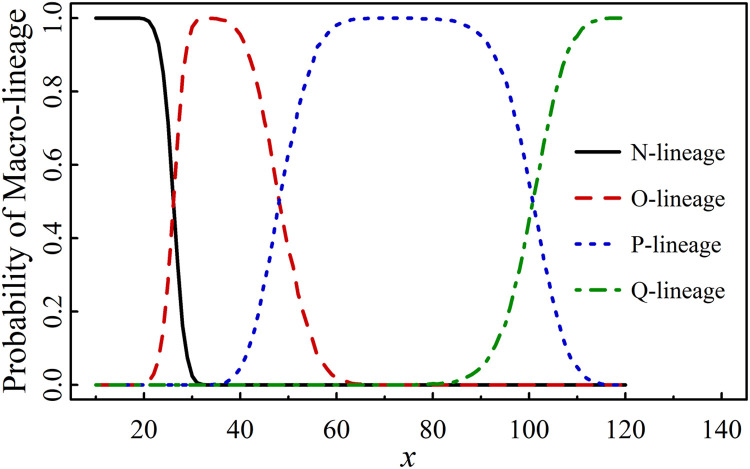
PML(Probability of Macro-lineage) vs *x* for 70 mutants (a = 153) given in Table S3.

At the subsequent time point *T*_3_ (January 4, 2025), after incorporating 70 variants from Table S3 into set *A*, the value of a increased to 153. The probabilities for each *j*-lineage (j = *N*, O, P, Q) based on these 70 mutants are shown in Fig. 4. The corresponding threshold values of *x* are defined as follows:

New*_x_*_._*_y_* ϵ N when *x* ≤ 21; New*_x_*_._*_y_* ϵ N or O when 21<x ≤ 30; New*_x_*_._*_y_* ϵ O when 30<x ≤ 37; New*_x_*_._*_y_* ϵ O or P when 37<x ≤ 61; New*_x_*_._*_y_* ϵ P when 61<*x* ≤ 84; New*_x.y_* ϵ P or Q when 84<x ≤ 112 and New*_x.y_* ϵ Q when *x* > 112.

#### 3.3.2 Law for the emergence of novel macro-lineages

Set *A*(*a*) is an observable set dependent on time *t*. Set *X* represents a set of assumed mutated sites, also time-dependent, which is used to predict new macro-lineages. We define *x*_dem1_ as the threshold value of *x* at which a new macro-lineage emerges with a nonzero probability (greater than 1 % in our calculations), and *x*_dem2_ as the threshold for its definitive occurrence, where a 99th percentile threshold was applied (shown in Fig. 2, Fig. 3, Fig. 4). Both *x*_dem1_ and *x*_dem2_ are functions of time *t*.

At time *tₖ*, the value of *a* is denoted as *aₖ*, with examples including *a*_1_ = 104, *a*_2_ = 128 and *a*_3_ = 153. The value of *x*_dem1_ for macro-lineage *j* (where j = *O*, P, Q) at time *tₖ* is denoted as *x*(*j*) dem1(*t_k_*), and similarly, *x*_dem2_ for macro-lineage *j* at time *tₖ* is denoted as *x*(*j*) dem2(*t_k_*). The values of *x*(*j*) dem1(*t_k_*) and *x*(*j*) dem2(*t_k_*) are presented in Fig. 2, Fig. 3, Fig. 4 and summarized in Table 1.

**Table 1:** Demarcation values *x*_dem1_ and *x*_dem2_ at three times *t*_1_ and *t*_2_ and *t*_3_.

*k*	*a_k_*	*x*(O) dem1(*t_k_*)	*x*(O) dem2(*t_k_*)	*x*(P) dem1(*t_k_*)	*x*(P) dem2(*t_k_*)	*x*(Q) dem1(*t_k_*)	*x*(Q) dem2(*t_k_*)
1	104	19	30	37	70	–	–
2	128	19	30	40	63	73	100
3	153	22	31	38	62	85	113

Note: *t*_1_<*t*_2_<*t*_3_. *t*_1_ is the time period later than *T*_1_=October 15, 2023, *t*_2_ is the time period later than *T*_2_= July 20, 2024, *t*_3_ is the time period later than *T*_3_= January 4, 2025. The starting point for each time period and the linked actual variants in three time periods can be found in Supplementary Table S1, S2 and S3 respectively.

Table 1 outlines the occurrence pattern of novel macro-lineages: the O-lineage emerges at x ≈ 19–22 and definitively occurs at x ≈ 30–31; the P-lineage emerges at x ≈ 37–40 and definitively occurs at x ≈ 62–70; the Q-lineage emerges at x ≈ 73–85 and definitively occurs at x ≈ 100–113. The threshold values deduced across different time periods are highly consistent.

Based on Table 1 and Fig. 2, we predicted the emergence of the P-lineage at time *t*_1_. However, no P-lineage is observed in the cladogenetic tree for 25 mutants (see Fig. 1 and Supplementary Figure S1). The P-lineage appears in the cladogenetic tree only when the number of mutants reaches 36 or more. This suggests that our prediction preceded the actual observation and phylogenetic confirmation.

**Fig. 5:**
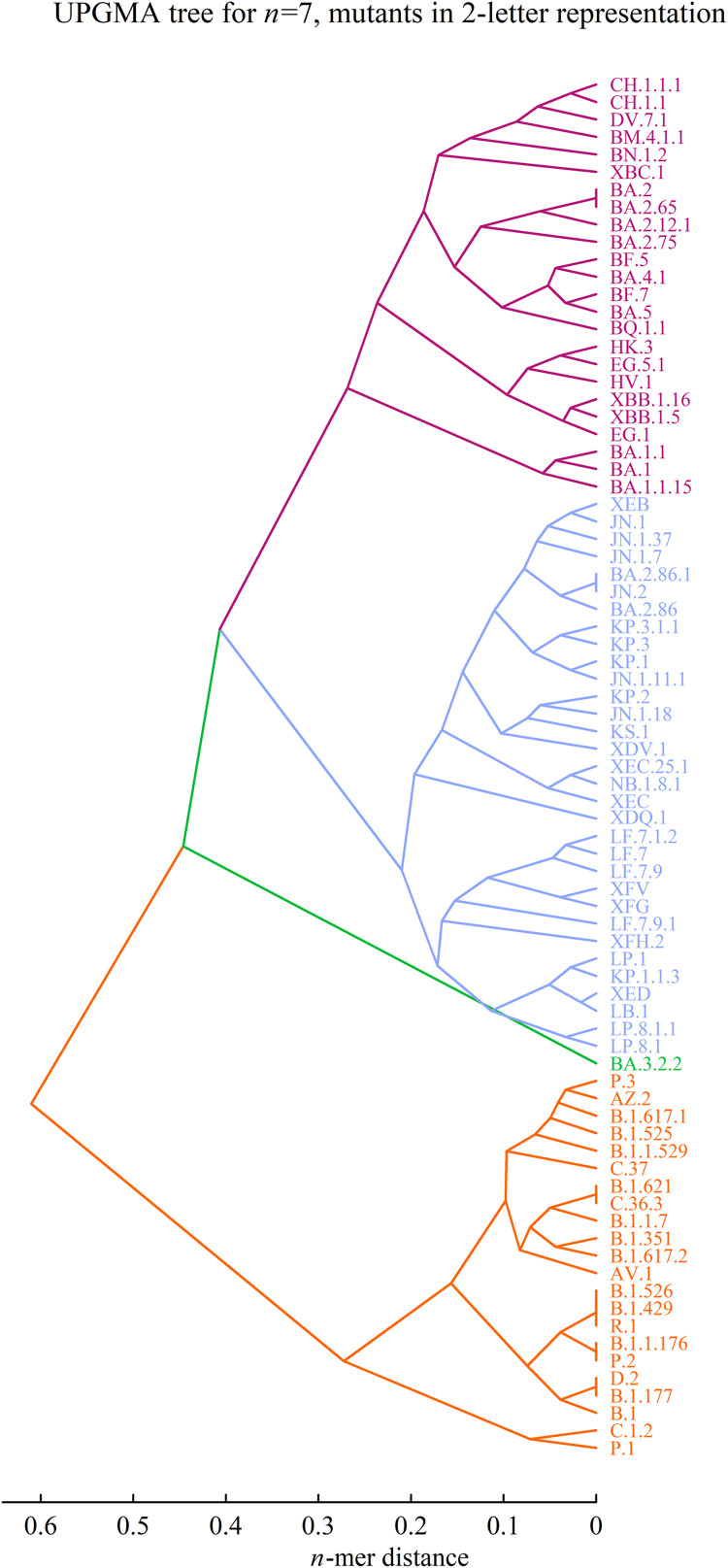
UPGMA tree of n = 7 in 2-letter representation for 79 mutants by taking new variants (in Next Strain, see Table S4) into account.

From Table 1 and Fig. 3, Fig. 4, we predicted the emergence of a new Q-lineage from the P-lineage at a sufficiently high *x* value (exceeding 73–85). Notably, the number of mutated sites (*x*) in the P-lineage never exceeded 68, as shown in Table S3. However, in Table S4, we identified a new variant, BA.3.2.2, with 74 mutated sites. To investigate whether BA.3.2.2 is associated with the emergence of a new lineage, we constructed a phylogenetic tree using a cladogenesis algorithm (Fig. 5).

Fig. 5 reveals that most new variants belong to the P-lineage, whereas BA.3.2.2 has diverged from it. Therefore, we hypothesize that the occurrence of BA.3.2.2 in April 2025 may signal the emergence of the new Q-lineage. To assess the reliability of this prediction method, we conducted retrospective testing (backtesting) and demonstrated that the model successfully predicted the emergence of the Omicron (O) lineage using only pre-2021 sequence data.

In conclusion, by conceptualizing stochastic mutation as a mechanism driving the emergence of new SARS-CoV-2 variants, we derived a principle governing macro-lineage transformation from N to P and then to Q. This approach offers a predictive framework for anticipating the future dominance of novel SARS-CoV-2 variants.

### 3.4 Each macro-lineage of SARS-CoV-2 has a specific survival time

#### 3.4.1 The relationship between the number of mutated sites and time t is a discontinuous function

**Fig. 6:**
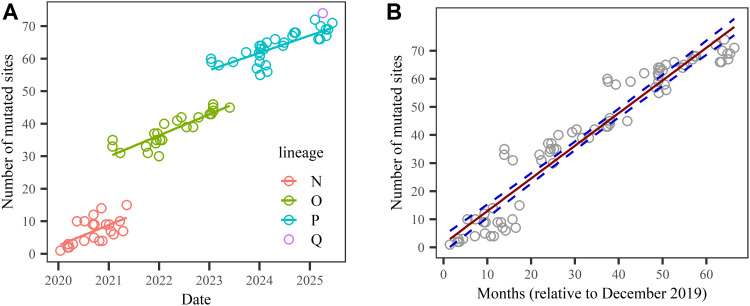
(A) Number of mutated sites versus the initial sample collection date. (B) Linear regression analysis of mutated site counts against initial sample collection dates, with 95 % confidence intervals (represented by blue dotted lines) calculated using linear regression theory.

According to the retrieval of SARS-CoV-2 variant data in Table S3, Table S5 presents the relationship between the number of mutated sites (NMS) in the spike protein and the initial global sample collection dates for each SARS-CoV-2 variant. We investigated the relationship and explored the potential evolutionary clock defined by the number of mutated sites (NMS) in the spike protein. Although mutation accumulation is influenced by founder effects and immune escape fitness plateaus, and mutation impact weighting (e.g., functional category or ΔΔG on ACE2 binding), we found that the NMS in the spike protein across variants can be ordered chronologically based on the dates of the first global sample collection. This suggests the existence of an approximate evolutionary clock. Further, our analysis reveals that NMS(*t*) represents a discontinuous function of time, corresponding to three distinct macro-lineages (Fig. 6A). This figure is derived directly from the data in Table S5.

#### 3.4.2 Linear regression of mutated sites vs. sample collection date as an approximation

We performed linear regression to analyze the relationship between the number of mutated sites and the initial sample collection date; the results are shown in Fig. 6B The linear model effectively captures the increasing trend in mutated sites over viral evolution. The regression yielded an *R*² value of 0.92 and an RSE (Residual Standard Error) of 6.68. Furthermore, using linear regression theory, we estimated a slope of 1.163 per month (95 % CI: ±0.076). Given the strong fit of this linear model, we will utilize the relationship between the number of mutated sites (*x*) and collection time (*t*) to predict the emergence of new viral lineages.

Given that viral mutation accumulation rates are non-uniform, replacing linear regression with a piecewise or segmented model would result in Fig. 6B being superseded by Fig. 6A. In this model, the relationship between the number of mutated sites (NMS) and time (*t*) is discontinuous, exhibiting stepwise changes at lineage transition points. However, the rate of NMS increase varies between adjacent lineages, with newer lineages typically demonstrating slower accumulation rates compared to older ones. This compensatory mechanism offsets the stepwise changes during lineage transitions, yielding an overall approximately linear relationship between NMS and emergence time. Our predictions for new lineages are based on extrapolating this linear trend over extended timeframes.

#### 3.4.3 Prediction of the emergence time of the possible Q macro-lineage

Based on the above analyses, we propose an algorithm for predicting the emergence timeline of macro-lineages. In the A–X model, the number of randomly mutated sites for a variant is denoted as *x*. Notably, NMS(*t*), which quantifies the accumulation of mutated sites over time, is intrinsically related to *x*. Using the data from Fig. 4—which shows the relationship between the probability of macro-lineage emergence (PML) and *x*, as well as the time dependence of NMS(*t*)—we predict the emergence time of the new macro-lineage Q. By extrapolating the linear trend over a longer period and applying linear regression, we estimate that the number of mutated sites will reach NMS =73.4 ± 2.6 to 85 ± 3 between the 62nd and 72nd months, and 100 ± 4 to 113 ± 5 between the 85th and 96th months, starting from December 2019. Combining these values with the thresholds *x*_dem1_ = 73–85 (indicating the initial emergence of Q) and *x*_dem2_ = 100–113 (indicating a major outbreak of Q), we preliminarily predict that macro-lineage Q will emerge around February to December 2025, and may experience a strong outbreak approximately 22 to 24 months later. Currently, the new variant BA.3.2.2, which emerged in April 2025, appears to signal the initial rise of macro-lineage Q (Fig. 5). However, since the mutation probabilities of sites on the spike protein are strongly influenced by diverse biological driving forces, the actual timeline for macro-lineage emergence may differ. Thus, the above estimation should be used for reference only.

## 4 Conclusions

1. Using the cladogram algorithm and the A–X model (Luo and Lv, 2024)—where Set *A* comprises existing mutated sites and Set *X* contains *x* randomly generated sites on the spike protein—we can investigate the relationship between stochastic mutations and the generation of new strains within a cladogram framework.

2. Based on the understanding that stochastic mutation drives the emergence of new SARS-CoV-2 variants, we deduced a principle governing macro-lineage transformation. By expanding the scope of stochastic sampling, we demonstrate that a new macro-lineage emerges alongside existing lineages once the number of randomly generated sites (*x*) reaches a critical threshold. As *x* increases further, the relative proportions of the four macro-lineages shift: O surpasses N, P subsequently surpasses O, and finally, a new lineage Q may emerge.

3. Based on the linear regression of the number of mutated sites per variant against its global initial sampling time, we propose an algorithm for predicting the emergence timeline of macro-lineages. By incorporating the influence of diverse biological drivers on spike protein mutation probabilities, this algorithm facilitates more accurate predictions of the actual emergence timing of new macro-lineages.

## Funding

This research did not receive any specific grant from funding agencies in the public, commercial, or not-for-profit sectors.

## Author statement

We, the authors of the manuscript titled “Stochastic Mutation as a Mechanism for the Emergence of SARS-CoV-2 New Variants”, declare that the data, methods, and conclusions presented in our submission to Virus Research have not been published in any peer-reviewed journal. The content of the paper does not involve any ethical or moral issues. Furthermore, there are no conflicts of interest among the authors.

## CRediT authorship contribution statement

**Liaofu Luo:** Writing – review & editing, Writing – original draft, Methodology, Investigation, Conceptualization. **Jun Lv:** Writing – review & editing, Visualization, Validation, Methodology, Investigation, Data curation, Conceptualization.

## Declaration of competing interest

The authors declare that they have no known competing financial interests or personal relationships that could have appeared to influence the work reported in this paper.

## Data Availability

Data will be made available on request.

## Appendix Supplementary materials

Image, application 1
