# Supplementary material for: Stochastic mutation as a mechanism for the emergence of SARS-CoV-2 new variants
Source: Virus Res. 2025 Nov 20;362:199667. doi: 10.1016/j.virusres.2025.199667 (PMC12789824; doi:10.1016/j.virusres.2025.199667)
Supplement: Supplementary file 1 [file mmc1.docx]

**Supplementary Material:**

**Stochastic Mutation as a Mechanism for the Emergence of SARS-CoV-2 New Variants**

Liaofu Luo^1*^, Jun Lv^2*^

^1^Faculty of Physical Science and Technology, Inner Mongolia University, 235 West College Road, Hohhot 010021, PR China

^2^College of Science, Inner Mongolia University of Technology, 49 Aymin Street, Hohhot 010051, PR China

^*^Correspondence:

Liaofu Luo

[lolfcm@imu.edu.cn](mailto:lolfcm@imu.edu.cn)

Jun Lv

[lujun@imut.edu.cn](mailto:lujun@imut.edu.cn)

**Supplementary Material 1：Mutation data of SARS-CoV-2 Variants**

**Table S1 Partial table of 25 variants and their mutation sites**

A total of 25 variants from the mutation reports provided by outbreak.info (<https://outbreak.info/>, accessed on 15 October 2023)

| **macro-lineage** | **No.** | **variant** | **NMS** | **mutated sites** |
| --- | --- | --- | --- | --- |
| N-lineage | 1 | P.2(Zeta) | 3 | **484**,614,1176 |
|  | 2 | B.1.1.7(Alpha) | 10 | 69,70,144,**501**,570,614,681,716,982,1118 |
|  | 3 | B.1.429(Epsilon) | 4 | 13,152,**452**,614 |
|  | 4 | B.1.351(Beta) | 10 | 80,215,241,242,243,**417**,**484**,**501**,614,701 |
|  | 5 | B.1.617.2(Delta) | 9 | 19,156,157,158,**452**,**478**,614,681,950 |
|  | 6 | P.1(Gamma) | 12 | 18,20,26,138,190,**417**,**484**,**501**,614,655,1027,1176 |
|  | 7 | B.1.617.1(Kappa) | 5 | **452**,**484**,614,681,1071 |
|  | 8 | B.1.621(Mu) | 9 | 95,144,145,**346**,**484**,**501**,614,681,950 |
|  | 9 | C.37(Lambda) | 14 | 75,76,246,247,248,249,250,251,252,253,**452**,**490**,614,859 |
|  | 10 | B.1.526(Iota) | 4 | 5,95,253,614 |
|  | 11 | B.1.525(Eta) | 9 | 52,67,69,70,144,**484**,614,677,888 |
|  | 12 | P.3(Theta) | 7 | **484**,**501**,614,681,1092,1101,1176 |
| O-lineage | 13 | BA.1 | 33 | 67,69,70,95,142,143,144,145,211,212,**339,371,373,375,477,478,484,493,496,498,501,505**,547,614,655,679,681,764,796,856,954,969,981 |
|  | 14 | BA.2 | 31 | 19,24,25,26,27,142,213,**339,371,373,375,376,405,408,417,440,477,478,484,493,498,501,505**,614,655,679,681,764,796,954,969 |
|  | 15 | BA.2.12.1 | 33 | 19,24,25,26,27,142,213,**339,371,373,375,376,405,408,417,440,452,477,478,484,493,498,501,505**,614,655,679,681,704,764,796,954,969 |
|  | 16 | BA.5 | 34 | 19,24,25,26,27,69,70,142,213,**339,371,373,375,376,405,408,417,440,452,477,478,484,486,498,501,505**,614,655,679,681,764,796,954,969 |
|  | 17 | BA.4.1 | 35 | 3,19,24,25,26,27,69,70,142,213,**339,371,373,375,376,405,408,417,440,452,477,478,484,486,498,501,505**,614,655,679,681,764,796,954,969 |
|  | 18 | BQ.1.1 | 37 | 19,24,25,26,27,69,70,142,213,**339,346,371,373,375,376,405,408,417,440,444,452,460,477,478,484,486,498,501,505**,614,655,679,681,764,796,954,969 |
|  | 19 | BA.2.75 | 30 | 19,24,210,213,257,**339**,**371**,**373**,**375**,**376**,**405**,**408**,**417**,**440**,**446**,**460**,**477**,**478**,**484**,**498**,**501**,**505**,614,655,679,681,764,796,954,969 |
|  | 20 | BF.7 | 35 | 19,24,25,26,27,69,70,142,213,**339,346,371,373,375,376,405,408,417,440,452,477,478,484,486,498,501,505**,614,655,679,681,764,796,954,969 |
|  | 21 | CH.1.1 | 41 | 19,24,25,26,27,142,147,152,157,210,213,257,**339,346,371,373,375,376,405,408,417,440,444,446,452,460,477,478,484,486,498,501,505**,614,655,679,681,764,796,954,969 |
|  | 22 | XBB.1.5 | 42 | 19,24,25,26,27,83,142,144,146,183,213,252,**339,346,368,371,373,375,376,405,408,417,440,445,446,460,477,478,484,486,490,498,501,505**,614,655,679,681,764,796,954,969 |
|  | 23 | XBB.1.16 | 43 | 19,24,25,26,27,83,142,144,146,180,183,213,252,**339,346,368,371,373,375,376,405,408,417,440,445,446,460,477,478,484,486,490,498,501,505**,614,655,679,681,764,796,954,969 |
|  | 24 | EG.1 | 43 | 19,24,25,26,27,83,142,144,146,183,213,252,**339,346,368,371,373,375,376,405,408,417,440,445,446,460,477,478,484,486,490,498,501,505**,613,614,655,679,681,764,796,954,969 |
|  | 25 | EG.5.1 | 44 | 19,24,25,26,27,52,83,142,144,146,183,213,252,**339,346,368,371,373,375,376,405,408,417,440,445,446,456,460,477,478,484,486,490,498,501,505**,614,655,679,681,764,796,954,969 |
| total |  |  | 104 | 3, 5, 13, 18, 19, 20, 24, 25, 26, 27, 52, 67, 69, 70, 75, 76, 80, 83, 95, 138, 142, 143, 144, 145, 146, 147, 152, 156, 157, 158, 180, 183, 190, 210, 211, 212, 213, 215, 241, 242, 243, 246, 247, 248, 249, 250, 251, 252, 253, 257, **339, 346, 368, 371, 373, 375, 376, 405, 408, 417, 440, 444, 445, 446, 452, 456, 460, 477, 478, 484, 486, 490, 493, 496, 498, 501, 505**, 547, 570, 613, 614, 655, 677, 679, 681, 701, 704, 716, 764, 796, 856, 859, 888, 950, 954, 969, 981, 982, 1027, 1071, 1092, 1101, 1118, 1176 |

NMS: number of mutated sites; Mutated sites are considered when they occur in at least 75% of the SARS-CoV-2 lineage sequences. Mutations in the spike protein's receptor-binding domain (RBD) are indicated in bold.

**Table S2 Partial table of 36 variants and their mutation sites**

A total of 36 variants from the mutation reports provided by outbreak.info (<https://outbreak.info/>, accessed on 20 July 2024)

| **macro-lineage** | **No.** | **variant** | **NMS** | **mutated sites** |
| --- | --- | --- | --- | --- |
| N-lineage | 1 | P.2(Zeta) | 3 | **484**,614,1176 |
|  | 2 | B.1.1.7(Alpha) | 10 | 69,70,144,**501**,570,614,681,716,982,1118 |
|  | 3 | B.1.429(Epsilon) | 4 | 13,152,**452**,614 |
|  | 4 | B.1.351(Beta) | 10 | 80,215,241,242,243,**417**,**484**,**501**,614,701 |
|  | 5 | B.1.617.2(Delta) | 9 | 19,156,157,158,**452**,**478**,614,681,950 |
|  | 6 | P.1(Gamma) | 12 | 18,20,26,138,190,**417**,**484**,**501**,614,655,1027,1176 |
|  | 7 | B.1.617.1(Kappa) | 5 | **452**,**484**,614,681,1071 |
|  | 8 | B.1.621(Mu) | 9 | 95,144,145,**346**,**484**,**501**,614,681,950 |
|  | 9 | C.37(Lambda) | 14 | 75,76,246,247,248,249,250,251,252,253,**452**,**490**,614,859 |
|  | 10 | B.1.526(Iota) | 4 | 5,95,253,614 |
|  | 11 | B.1.525(Eta) | 9 | 52,67,69,70,144,**484**,614,677,888 |
|  | 12 | P.3(Theta) | 7 | **484**,**501**,614,681,1092,1101,1176 |
| O-lineage | 13 | BA.1 | 33 | 67,69,70,95,142,143,144,145,211,212,**339,371,373,375,477,478,484,493,496,498,501,505**,547,614,655,679,681,764,796,856,954,969,981 |
|  | 14 | BA.2 | 31 | 19,24,25,26,27,142,213,**339,371,373,375,376,405,408,417,440,477,478,484,493,498,501,505**,614,655,679,681,764,796,954,969 |
|  | 15 | BA.2.12.1 | 33 | 19,24,25,26,27,142,213,**339,371,373,375,376,405,408,417,440,452,477,478,484,493,498,501,505**,614,655,679,681,704,764,796,954,969 |
|  | 16 | BA.5 | 34 | 19,24,25,26,27,69,70,142,213,**339,371,373,375,376,405,408,417,440,452,477,478,484,486,498,501,505**,614,655,679,681,764,796,954,969 |
|  | 17 | BA.4.1 | 35 | 3,19,24,25,26,27,69,70,142,213,**339,371,373,375,376,405,408,417,440,452,477,478,484,486,498,501,505**,614,655,679,681,764,796,954,969 |
|  | 18 | BQ.1.1 | 37 | 19,24,25,26,27,69,70,142,213,**339,346,371,373,375,376,405,408,417,440,444,452,460,477,478,484,486,498,501,505**,614,655,679,681,764,796,954,969 |
|  | 19 | BA.2.75 | 30 | 19,24,210,213,257,**339**,**371**,**373**,**375**,**376**,**405**,**408**,**417**,**440**,**446**,**460**,**477**,**478**,**484**,**498**,**501**,**505**,614,655,679,681,764,796,954,969 |
|  | 20 | BF.7 | 35 | 19,24,25,26,27,69,70,142,213,**339,346,371,373,375,376,405,408,417,440,452,477,478,484,486,498,501,505**,614,655,679,681,764,796,954,969 |
|  | 21 | CH.1.1 | 41 | 19,24,25,26,27,142,147,152,157,210,213,257,**339,346,371,373,375,376,405,408,417,440,444,446,452,460,477,478,484,486,498,501,505**,614,655,679,681,764,796,954,969 |
|  | 22 | XBB.1.5 | 42 | 19,24,25,26,27,83,142,144,146,183,213,252,**339,346,368,371,373,375,376,405,408,417,440,445,446,460,477,478,484,486,490,498,501,505**,614,655,679,681,764,796,954,969 |
|  | 23 | XBB.1.16 | 43 | 19,24,25,26,27,83,142,144,146,180,183,213,252,**339,346,368,371,373,375,376,405,408,417,440,445,446,460,477,478,484,486,490,498,501,505**,614,655,679,681,764,796,954,969 |
|  | 24 | EG.1 | 43 | 19,24,25,26,27,83,142,144,146,183,213,252,**339,346,368,371,373,375,376,405,408,417,440,445,446,460,477,478,484,486,490,498,501,505**,613,614,655,679,681,764,796,954,969 |
|  | 25 | EG.5.1 | 44 | 19,24,25,26,27,52,83,142,144,146,183,213,252,**339,346,368,371,373,375,376,405,408,417,440,445,446,456,460,477,478,484,486,490,498,501,505**,614,655,679,681,764,796,954,969 |
|  | 26 | HV.1 | 46 | 19,24,25,26,27,52,83,142,144,146,157,183,213,252,**339,346,368,371,373,375,376,405,408,417,440,445,446,452,456,460,477,478,484,486,490,498,501,505**,614,655,679,681,764,796,954,969 |
| P-lineage | 27 | JN.1 | 60 | 19,21,24,25,26,27,50,69,70,127,142,144,157,158,211,212,213,216,245,264,**332,339,356,371,373,375,376,403,405,408,417,440,445,446,450,452,455,460,477,478,481,483,484,486,498,501,505**,554,570,614,621,655,679,681,764,796,939,954,969,1143 |
|  | 28 | BA.2.86.1 | 59 | 19,21,24,25,26,27,50,69,70,127,142,144,157,158,211,212,213,216,245,264,**332,339,356,371,373,375,376,403,405,408,417,440,445,446,450,452,460,477,478,481,483,484,486,498,501,505**,554,570,614,621,655,679,681,764,796,939,954,969,1143 |
|  | 29 | BA.2.86 | 58 | 19,21,24,25,26,27,50,69,70,127,142,144,157,158,211,212,213,216,245,264,**332,339,356,371,373,375,376,403,405,408,417,440,445,446,450,452,460,477,478,481,484,486,498,501,505**,554,570,614,621,655,679,681,764,796,939,954,969,1143 |
|  | 30 | JN.1.7 | 62 | 19,21,24,25,26,27,50,69,70,127,142,144,157,158,211,212,213,216,245,264,**332,339,356,371,373,375,376,403,405,408,417,440,445,446,450,452,455,460,477,478,481,483,484,486,498,501,505**,554,570,572,614,621,655,679,681,764,796,939,954,969,1143,1150 |
|  | 31 | KP.3.1.1 | 64 | 19,21,24,25,26,27,31,50,69,70,127,142,144,157,158,211,212,213,216,245,264,**332,339,356,371,373,375,376,403,405,408,417,440,445,446,450,452,455,456,460,477,478,481,483,484,486,493,498,501,505**,554,570,614,621,655,679,681,764,796,939,954,969,1104,1143 |
|  | 32 | KP.2 | 59 | 19,21,50,69,70,127,142,144,157,158,211,212,213,216,245,264,**332,339,346,356,371,373,375,376,403,405,408,417,440,445,446,450,452,455,456,460,477,478,481,483,484,486,498,501,505**,554,570,614,621,655,679,681,764,796,939,954,969,1104,1143 |
|  | 33 | JN.1.37 | 61 | 19,21,24,25,26,27,50,69,70,127,142,144,157,158,211,212,213,216,245,264,**332,339,356,371,373,375,376,403,405,408,417,440,445,446,450,452,455,460,477,478,481,483,484,486,498,501,505**,554,570,614,621,655,679,680,681,764,796,939,954,969,1143 |
|  | 34 | XDQ.1 | 55 | 19,21,24,25,26,27,50,69,70,127,142,144,157,158,211,212,213,216,245,264,**332,339,356,371,373,375,376,403,405,408,417,440,445,446,450,452,460,475,483,484,486,498,501,505**,554,570,614,621,655,679,681,764,796,954,969 |
|  | 35 | LB.1 | 64 | 19,21,24,25,26,27,31,50,69,70,127,142,144,157,158,183,211,212,213,216,245,264,**332,339,346,356,371,373,375,376,403,405,408,417,440,445,446,450,452,455,456,460,477,478,481,483,484,486,498,501,505**,554,570,614,621,655,679,681,764,796,939,954,969,1143 |
|  | 36 | KP.1 | 63 | 19,21,24,25,26,27,50,69,70,127,142,144,157,158,211,212,213,216,245,264,**332,339,356,371,373,375,376,403,405,408,417,440,445,446,450,452,455,456,460,477,478,481,483,484,486,498,501,505**,554,570,614,621,655,679,681,764,796,939,954,969,1086,1104,1143 |
| total |  |  | 128 | 3,5,13,18,19,20,21,24,25,26,27,31,50,52,67,69,70,75,76,80,83,95,127,138,142,143,144,145,146,147,152,156,157,158,180,183,190,210,211,212,213,215,216,241,242,243,245,246,247,248,249,250,251,252,253,257,264,**332,339,346,356,368,371,373,375,376,403,405,408,417,440,444,445,446,450,452,455,456,460,475,477,478,481,483,484,486,490,493,496,498,501,505**,547,554,570,572,613,614,621,655,677,679,680,681,701,704,716,764,796,856,859,888,939,950,954,969,981,982,1027,1071,1086,1092,1101,1104,1118,1143,1150,1176 |

NMS: number of mutated sites; Mutated sites are considered when they occur in at least 75% of the SARS-CoV-2 lineage sequences. Mutations in the spike protein's receptor-binding domain (RBD) are indicated in bold.

**Table S3** General table of 70 variants and their mutation sites

A total of 70 variants from the mutation reports provided by outbreak.info (<https://outbreak.info/>, accessed on 4 January 2025)

| **macro-lineage** | **No.** | **variant** | **NMS** | **mutated sites** |
| --- | --- | --- | --- | --- |
| N-lineage | 1 | B.1 | 1 | 614 |
|  | 2 | B.1.177 | 2 | 222,614 |
|  | 3 | B.1.1.176 | 3 | 20,**357**,614 |
|  | 4 | D.2 | 2 | **477**,614 |
|  | 5 | P.2(Zeta) | 3 | **484**,614,1176 |
|  | 6 | B.1.1.7(Alpha) | 10 | 69,70,144,**501**,570,614,681,716,982,1118 |
|  | 7 | B.1.429(Epsilon) | 4 | 13,152,**452**,614 |
|  | 8 | B.1.351(Beta) | 10 | 80,215,241,242,243,**417**,**484**,**501**,614,701 |
|  | 9 | B.1.617.2(Delta) | 9 | 19,156,157,158,**452**,**478**,614,681,950 |
|  | 10 | P.1(Gamma) | 12 | 18,20,26,138,190,**417**,**484**,**501**,614,655,1027,1176 |
|  | 11 | B.1.617.1(Kappa) | 5 | **452**,**484**,614,681,1071 |
|  | 12 | B.1.621(Mu) | 9 | 95,144,145,**346**,**484**,**501**,614,681,950 |
|  | 13 | R.1 | 4 | 152,**484**,614,769 |
|  | 14 | C.37(Lambda) | 14 | 75,76,246,247,248,249,250,251,252,253,**452**,**490**,614,859 |
|  | 15 | B.1.526(Iota) | 4 | 5,95,253,614 |
|  | 16 | B.1.525(Eta) | 9 | 52,67,69,70,144,**484**,614,677,888 |
|  | 17 | C.36.3 | 9 | 12,69,70,152,**346**,**452**,614,677,899 |
|  | 18 | P.3(Theta) | 7 | **484**,**501**,614,681,1092,1101,1176 |
|  | 19 | AZ.2 | 6 | 95,144,**484**,614,681,796 |
|  | 20 | AV.1 | 10 | 80,95,142,144,**439**,**484**,614,681,1130,1139 |
|  | 21 | B.1.1.529 | 7 | **373**,**478**,614,655,679,681,954 |
|  | 22 | C.1.2 | 15 | 9,136,144,190,215,243,244,**449**,**484**,**501**,614,655,679,716,859 |
| O-lineage | 23 | BA.1 | 33 | 67,69,70,95,142,143,144,145,211,212,**339,371,373,375,477,478,484,493,496,498,501,505**,547,614,655,679,681,764,796,856,954,969,981 |
|  | 24 | BA.1.1 | 35 | 67,69,70,95,142,143,144,145,211,212,**339,346,371,373,375,446,477,478,484,493,496,498,501,505**,547,614,655,679,681,764,796,856,954,969,981 |
|  | 25 | BA.2 | 31 | 19,24,25,26,27,142,213,**339,371,373,375,376,405,408,417,440,477,478,484,493,498,501,505**,614,655,679,681,764,796,954,969 |
|  | 26 | BA.2.12.1 | 33 | 19,24,25,26,27,142,213,**339,371,373,375,376,405,408,417,440,452,477,478,484,493,498,501,505**,614,655,679,681,704,764,796,954,969 |
|  | 27 | BA.2.65 | 31 | 19,24,25,26,27,142,213,**339,371,373,375,376,405,408,417,440,477,478,484,493,498,501,505**,614,655,679,681,764,796,954,969 |
|  | 28 | BA.1.1.15 | 37 | 67,69,70,95,142,143,144,145,211,212,**339,346,371,373,375,417,440,446,477,478,484,493,496,498,501,505**,547,614,655,679,681,764,796,856,954,969,981 |
|  | 29 | BA.5 | 34 | 19,24,25,26,27,69,70,142,213,**339,371,373,375,376,405,408,417,440,452,477,478,484,486,498,501,505**,614,655,679,681,764,796,954,969 |
|  | 30 | BA.4.1 | 35 | 3,19,24,25,26,27,69,70,142,213,**339,371,373,375,376,405,408,417,440,452,477,478,484,486,498,501,505**,614,655,679,681,764,796,954,969 |
|  | 31 | BQ.1.1 | 37 | 19,24,25,26,27,69,70,142,213,**339,346,371,373,375,376,405,408,417,440,444,452,460,477,478,484,486,498,501,505**,614,655,679,681,764,796,954,969 |
|  | 32 | BA.2.75 | 30 | 19,24,210,213,257,**339**,**371**,**373**,**375**,**376**,**405**,**408**,**417**,**440**,**446**,**460**,**477**,**478**,**484**,**498**,**501**,**505**,614,655,679,681,764,796,954,969 |
|  | 33 | BF.5 | 35 | 19,24,25,26,27,69,70,142,213,**339,371,373,375,376,405,408,417,440,452,477,478,484,486,498,501,505**,614,655,679,681,764,796,954,969,1020 |
|  | 34 | BF.7 | 35 | 19,24,25,26,27,69,70,142,213,**339,346,371,373,375,376,405,408,417,440,452,477,478,484,486,498,501,505**,614,655,679,681,764,796,954,969 |
|  | 35 | BN.1.2 | 40 | 19,24,25,26,27,142,147,152,157,210,213,257,**339,346,356,371,373,375,376,405,408,417,440,446,460,477,478,484,490,498,501,505**,614,655,679,681,764,796,954,969 |
|  | 36 | CH.1.1 | 41 | 19,24,25,26,27,142,147,152,157,210,213,257,**339,346,371,373,375,376,405,408,417,440,444,446,452,460,477,478,484,486,498,501,505**,614,655,679,681,764,796,954,969 |
|  | 37 | XBB.1.5 | 42 | 19,24,25,26,27,83,142,144,146,183,213,252,**339,346,368,371,373,375,376,405,408,417,440,445,446,460,477,478,484,486,490,498,501,505**,614,655,679,681,764,796,954,969 |
|  | 38 | BM.4.1.1 | 39 | 19,24,25,26,27,142,147,152,157,210,213,257,**339,346,371,373,375,376,405,408,417,440,446,460,477,478,484,486,498,501,505**,614,655,679,681,764,796,954,969 |
|  | 39 | XBC.1 | 39 | 19,25,142,144,156,157,158,209,212,215,222,243,244,**371**,**373**,**375**,**376**,**405**,**408**,**417**,**440**,**446**,**452**,**477**,**478**,**484**,**486**,**498**,**501**,**505**,614,655,679,681,703,764,796,954,969 |
|  | 40 | CH.1.1.1 | 42 | 19,24,25,26,27,142,147,152,157,185,210,213,257,**339,346,371,373,375,376,405,408,417,440,444,446,452,460,477,478,484,486,498,501,505**,614,655,679,681,764,796,954,969 |
|  | 41 | XBB.1.16 | 43 | 19,24,25,26,27,83,142,144,146,180,183,213,252,**339,346,368,371,373,375,376,405,408,417,440,445,446,460,477,478,484,486,490,498,501,505**,614,655,679,681,764,796,954,969 |
|  | 42 | EG.1 | 43 | 19,24,25,26,27,83,142,144,146,183,213,252,**339,346,368,371,373,375,376,405,408,417,440,445,446,460,477,478,484,486,490,498,501,505**,613,614,655,679,681,764,796,954,969 |
|  | 43 | HV.1 | 46 | 19,24,25,26,27,52,83,142,144,146,157,183,213,252,**339,346,368,371,373,375,376,405,408,417,440,445,446,452,456,460,477,478,484,486,490,498,501,505**,614,655,679,681,764,796,954,969 |
|  | 44 | HK.3 | 45 | 19,24,25,26,27,52,83,142,144,146,183,213,252,**339,346,368,371,373,375,376,405,408,417,440,445,446,455,456,460,477,478,484,486,490,498,501,505**,614,655,679,681,764,796,954,969 |
|  | 45 | EG.5.1 | 44 | 19,24,25,26,27,52,83,142,144,146,183,213,252,**339,346,368,371,373,375,376,405,408,417,440,445,446,456,460,477,478,484,486,490,498,501,505**,614,655,679,681,764,796,954,969 |
|  | 46 | DV.7.1 | 45 | 19,24,25,26,27,142,147,152,157,185,210,213,257,**339,346,371,373,375,376,405,408,417,440,444,446,452,455,456,460,477,478,484,486,498,501,505**,614,655,679,681,764,796,858,954,969 |
| P-lineage | 47 | JN.1 | 60 | 19,21,24,25,26,27,50,69,70,127,142,144,157,158,211,212,213,216,245,264,**332,339,356,371,373,375,376,403,405,408,417,440,445,446,450,452,455,460,477,478,481,483,484,486,498,501,505**,554,570,614,621,655,679,681,764,796,939,954,969,1143 |
|  | 48 | BA.2.86.1 | 59 | 19,21,24,25,26,27,50,69,70,127,142,144,157,158,211,212,213,216,245,264,**332,339,356,371,373,375,376,403,405,408,417,440,445,446,450,452,460,477,478,481,483,484,486,498,501,505**,554,570,614,621,655,679,681,764,796,939,954,969,1143 |
|  | 49 | BA.2.86 | 58 | 19,21,24,25,26,27,50,69,70,127,142,144,157,158,211,212,213,216,245,264,**332,339,356,371,373,375,376,403,405,408,417,440,445,446,450,452,460,477,478,481,484,486,498,501,505**,554,570,614,621,655,679,681,764,796,939,954,969,1143 |
|  | 50 | JN.2 | 59 | 19,21,24,25,26,27,50,69,70,127,142,144,157,158,211,212,213,216,245,264,**332,339,356,371,373,375,376,403,405,408,417,440,445,446,450,452,460,477,478,481,483,484,486,498,501,505**,554,570,614,621,655,679,681,764,796,939,954,969,1143 |
|  | 51 | JN.1.7 | 62 | 19,21,24,25,26,27,50,69,70,127,142,144,157,158,211,212,213,216,245,264,**332,339,356,371,373,375,376,403,405,408,417,440,445,446,450,452,455,460,477,478,481,483,484,486,498,501,505**,554,570,572,614,621,655,679,681,764,796,939,954,969,1143,1150 |
|  | 52 | JN.1.18 | 57 | 19,21,50,69,70,127,142,144,157,158,211,212,213,216,245,264,**332,339,346,356,371,373,375,376,403,405,408,417,440,445,446,450,452,455,460,477,478,481,483,484,486,498,501,505**,554,570,614,621,655,679,681,764,796,939,954,969,1143 |
|  | 53 | JN.1.11.1 | 62 | 19,21,24,25,26,27,50,69,70,127,142,144,157,158,211,212,213,216,245,264,**332,339,356,371,373,375,376,403,405,408,417,440,445,446,450,452,455,456,460,477,478,481,483,484,486,498,501,505**,554,570,614,621,655,679,681,764,796,939,954,969,1104,1143 |
|  | 54 | KP.3.1.1 | 64 | 19,21,24,25,26,27,31,50,69,70,127,142,144,157,158,211,212,213,216,245,264,**332,339,356,371,373,375,376,403,405,408,417,440,445,446,450,452,455,456,460,477,478,481,483,484,486,493,498,501,505**,554,570,614,621,655,679,681,764,796,939,954,969,1104,1143 |
|  | 55 | KP.2 | 59 | 19,21,50,69,70,127,142,144,157,158,211,212,213,216,245,264,**332,339,346,356,371,373,375,376,403,405,408,417,440,445,446,450,452,455,456,460,477,478,481,483,484,486,498,501,505**,554,570,614,621,655,679,681,764,796,939,954,969,1104,1143 |
|  | 56 | JN.1.37 | 61 | 19,21,24,25,26,27,50,69,70,127,142,144,157,158,211,212,213,216,245,264,**332,339,356,371,373,375,376,403,405,408,417,440,445,446,450,452,455,460,477,478,481,483,484,486,498,501,505**,554,570,614,621,655,679,680,681,764,796,939,954,969,1143 |
|  | 57 | XEB | 61 | 19,21,24,25,26,27,50,69,70,127,142,144,157,158,211,212,213,216,245,264,**332,339,356,371,373,375,376,403,405,408,417,440,445,446,450,452,455,460,477,478,481,483,484,486,498,501,505**,554,570,614,621,655,679,681,764,796,939,954,969,1143,1174 |
|  | 58 | XDQ.1 | 55 | 19,21,24,25,26,27,50,69,70,127,142,144,157,158,211,212,213,216,245,264,**332,339,356,371,373,375,376,403,405,408,417,440,445,446,450,452,460,475,483,484,486,498,501,505**,554,570,614,621,655,679,681,764,796,954,969 |
|  | 59 | KP.3 | 63 | 19,21,24,25,26,27,50,69,70,127,142,144,157,158,211,212,213,216,245,264,**332,339,356,371,373,375,376,403,405,408,417,440,445,446,450,452,455,456,460,477,478,481,483,484,486,493,498,501,505**,554,570,614,621,655,679,681,764,796,939,954,969,1104,1143 |
|  | 60 | LB.1 | 64 | 19,21,24,25,26,27,31,50,69,70,127,142,144,157,158,183,211,212,213,216,245,264,**332,339,346,356,371,373,375,376,403,405,408,417,440,445,446,450,452,455,456,460,477,478,481,483,484,486,498,501,505**,554,570,614,621,655,679,681,764,796,939,954,969,1143 |
|  | 61 | KP.1 | 63 | 19,21,24,25,26,27,50,69,70,127,142,144,157,158,211,212,213,216,245,264,**332,339,356,371,373,375,376,403,405,408,417,440,445,446,450,452,455,456,460,477,478,481,483,484,486,498,501,505**,554,570,614,621,655,679,681,764,796,939,954,969,1086,1104,1143 |
|  | 62 | KS.1 | 58 | 50,59,69,70,127,142,144,157,158,211,212,213,216,245,264,**332,339,346,356,371,373,375,376,403,405,408,417,440,445,446,450,452,455,456,460,477,478,481,483,484,486,498,501,505**,554,570,614,621,655,679,681,764,796,939,954,969,1087,1143 |
|  | 63 | KP.1.1.3 | 65 | 19,21,24,25,26,27,31,50,69,70,127,142,144,157,158,211,212,213,216,245,264,**332,339,346,356,371,373,375,376,403,405,408,417,440,445,446,450,452,455,456,460,477,478,481,483,484,486,498,501,505**,554,570,614,621,655,679,681,764,796,939,954,969,1086,1104,1143 |
|  | 64 | XDV.1 | 56 | 19,21,50,69,70,127,142,144,157,158,211,212,213,216,245,264,**332,339,356,371,373,375,376,403,405,408,417,440,445,446,450,452,455,456,460,477,478,481,484,486,498,501,505**,554,570,614,621,655,679,681,764,796,939,954,969,1143 |
|  | 65 | LP.1 | 66 | 19,21,24,25,26,27,31,50,69,70,127,142,144,157,158,211,212,213,216,245,264,**332,339,346,356,371,373,375,376,403,405,408,417,440,445,446,450,452,455,456,460,477,478,481,483,484,486,498,501,505**,554,570,614,621,655,679,681,764,796,939,954,969,1086,1104,1143,1229 |
|  | 66 | XED | 64 | 19,21,24,25,26,27,31,50,69,70,127,142,144,157,158,211,212,213,216,245,264,**332,339,346,356,371,373,375,376,403,405,408,417,440,445,446,450,452,455,456,460,477,478,481,483,484,486,498,501,505**,554,570,614,621,655,679,681,764,796,939,954,969,1143,1263 |
|  | 67 | XEC | 65 | 19,21,22,24,25,26,27,50,59,69,70,127,142,144,157,158,211,212,213,216,245,264,**332,339,356,371,373,375,376,403,405,408,417,440,445,446,450,452,455,456,460,477,478,481,483,484,486,493,498,501,505**,554,570,614,621,655,679,681,764,796,939,954,969,1104,1143 |
|  | 68 | LF.7 | 67 | 19,21,22,24,25,26,27,31,50,69,70,127,142,144,157,158,182,190,211,212,213,216,245,264,**332,339,346,356,371,373,375,376,403,405,408,417,440,444,445,446,450,452,455,456,460,477,478,481,483,484,486,498,501,505**,554,570,614,621,655,679,681,764,796,939,954,969,1143 |
|  | 69 | LF.7.1.2 | 68 | 19,21,22,24,25,26,27,31,50,69,70,127,142,144,157,158,182,190,211,212,213,216,245,264,**332,339,346,356,371,373,375,376,403,405,408,417,440,444,445,446,450,452,455,456,460,475,477,478,481,483,484,486,498,501,505**,554,570,614,621,655,679,681,764,796,939,954,969,1143 |
|  | 70 | LP.8.1 | 68 | 19,21,24,25,26,27,31,50,69,70,127,142,144,157,158,186,190,211,212,213,216,245,264,**332,339,346,356,371,373,375,376,403,405,408,417,440,445,446,450,452,455,456,460,477,478,481,483,484,486,493,498,501,505**,554,570,614,621,655,679,681,764,796,939,954,969,1086,1104,1143 |
| total |  |  | 153 | 3,5,9,12,13,18,19,20,21,22,24,25,26,27,31,50,52,59,67,69,70,75,76,80,83,95,127,136,138,142,143,144,145,146,147,152,156,157,158,180,182,183,185,186,190,209,210,211,212,213,215,216,222,241,242,243,244,245,246,247,248,249,250,251,252,253,257,264,**332,339,346,356,357,368,371,373,375,376,403,405,408,417,439,440,444,445,446,449,450,452,455,456,460,475,477,478,481,483,484,486,490,493,496,498,501,505**,547,554,570,572,613,614,621,655,677,679,680,681,701,703,704,716,764,769,796,856,858,859,888,899,939,950,954,969,981,982,1020,1027,1071,1086,1087,1092,1101,1104,1118,1130,1139,1143,1150,1174,1176,1229,1263 |

NMS: number of mutated sites; Mutated sites are considered when they occur in at least 75% of the SARS-CoV-2 lineage sequences. Mutations in the spike protein's receptor-binding domain (RBD) are indicated in bold.

**Note:** 70 variants written in red are taken from the mutation reports provided by non- VOC samples in outbreak.info. It leads to the total number of mutated sites from 147 (in original case of 63 variants) increased to 153.

**Table S4** New variants of SARS-CoV-2

A total of 9 variants from the mutation reports provided by NextStrain (https://nextstrain.org/ncov/gisaid/global/6m, accessed on 7 September 2025)

| **variant** | **NMS** | **Collection date** | **mutated sites** |
| --- | --- | --- | --- |
| XEC.25.1 | 66 | 2025/3/3 | 19,21,22,24,25,26,27,50,59,69,70,127,144,142,157,158,211,212,213,216,245,264,**332,339,356,371,373,375,376,403,405,408,417,435,440,445,446,450,452,455,456,460,477,478,481,483,484,486,493,498,501,505**,554,570,614,621,655,679,681,764,796,939,954,969,1104,1143 |
| NB.1.8.1 | 66 | 2025/3/15 | 19,21,22,24,25,26,27,50,59,69,70,127,142,144,157,158,184,211,212,213,216,245,264,**332,339,356,371,373,375,376,403,405,408,417,435,440,445,446,450,452,455,456,460,477,478,481,483,484,486,493,498,501,505**,554,570,614,621,655,679,681,764,796,939,954,969,1143 |
| XFH.2 | 67 | 2025/4/30 | 19,21,22,24,25,26,27,31,50,69,70,127,142,144,157,158,182,190,213,216,245,264,**332,335,339,346,356,371,373,375,376,385,403,405,408,417,440,445,446,450,452,455,456,460,477,478,481,484,486,493,498,501,505**,554,570,614,621,655,677,679,681,764,796,939,954,969,1143 |
| LP.8.1.1 | 69 | 2025/4/26 | 5,19,21,24,25,26,27,31,50,69,70,127,142,144,157,158,186,190,211,212,213,216,245,264,**332,339,346,356,371,373,375,376,403,405,408,417,440,445,446,450,452,455,456,460,477,478,481,483,484,486,493,498,501,505**,554,570,614,621,655,679,681,764,796,939,954,969,1086,1104,1143 |
| LF.7.9 | 69 | 2025/5/13 | 19,21,22,24,25,26,27,31,50,69,70,127,142,144,157,158,182,190,211,212,213,216,245,264,**332,339,346,356,371,373,375,376,403,405,408,417,440,441,444,445,446,450,452,455,456,460,475,477,478,481,483,484,486,498,501,505**,554,570,614,621,655,679,681,764,796,939,954,969,1143 |
| XFG | 70 | 2025/3/20 | 19,21,22,24,25,26,27,31,50,69,70,127,142,144,157,158,182,190,211,212,213,216,245,264,**332,339,346,356,371,373,375,376,403,405,408,417,440,444,445,446,450,452,455,456,460,477,478,481,483,484,486,487,493,498,501,505**,554,570,572,614,621,655,679,681,764,796,939,954,969,1143 |
| XFV | 71 | 2025/6/12 | 19,21,22,24,25,26,27,31,50,69,70,127,142,144,157,158,182,190,211,212,213,216,245,264,**332,339,346,356,371,373,375,376,403,405,408,417,440,444,445,446,450,452,455,456,460,477,478,481,483,484,486,487,493,498,501,505**,554,570,572,614,621,655,679,680,681,764,796,939,954,969,1143 |
| LF.7.9.1 | 72 | 2025/2/6 | 17,18,19,20,21,22,23,24,25,26,27,31,50,69,70,127,142,144,157,158,182,190,211,212,213,216,245,264,**332,339,346,356,371,373,375,376,403,405,408,440,441,444,445,446,450,452,455,456,460,475,477,478,481,483,484,486,498,501,505**,554,570,614,621,655,679,681,764,796,939,954,969,1143 |
| BA.3.2.2 | 74 | 2025/4/4 | 9,21,26,67,69,70,95,101,136,137,138,139,140,141,142,143,144,145,146,147,157,164,172,187,211,212,242,243,251,326,**339,348,356,371,373,375,403,405,408,417,435,440,445,446,452,460,477,478,484,496,498,501,529**,554,575,583,614,625,641,642,654,655,679,681,688,704,764,795,796,852,939,954,969,1184 |

**Table S5 Retrieval of SARS-CoV-2 variant data in Table S3: The number of mutated sites (NMS) in the spike protein and the first global sample collection dates for each variant arranged in chronological order**

| **Macro-lineage** | **Variant** | **NMS** * | **Earliest date ^‡^** | **Variant** | **NMS** * | **Earliest date ^‡^** |
| --- | --- | --- | --- | --- | --- | --- |
| N-lineage | B.1 | 1 | 15 Jan 2020 | B.1.621 | 9 | 19 Sep 2020 |
|  | B.1.177 | 2 | 7 Mar 2020 | R.1 | 4 | 24 Oct 2020 |
|  | B.1.1.176 | 3 | 12 Mar 2020 | C.37 | 14 | 8 Nov 2020 |
|  | D.2 | 2 | 19 Mar 2020 | B.1.526 | 4 | 15 Nov 2020 |
|  | P.2 | 3 | 15 Apr 2020 | B.1.525 | 9 | 11 Dec 2020 |
|  | B.1.1.7 | 10 | 14 May 2020 | C.36.3 | 9 | 4 Jan 2021 |
|  | B.1.429 | 4 | 6 Jul 2020 | P.3 | 7 | 15 Jan 2021 |
|  | B.1.351 | 10 | 9 Jul 2020 | AZ.2 | 6 | 5 Feb 2021 |
|  | B.1.617.2 | 9 | 7 Sep 2020 | AV.1 | 10 | 23 Mar 2021 |
|  | P.1 | 12 | 11 Sep 2020 | B.1.1.529 | 7 | 15 Apr 2021 |
|  | B.1.617.1 | 5 | 15 Sep 2020 | C.1.2 | 15 | 11 May 2021 |
| O-lineage | BA.1 | 33 | 27 Jan 2021 | BN.1.2 | 40 | 7 Feb 2022 |
|  | BA.1.1 | 35 | 28 Jan 2021 | CH.1.1 | 41 | 12 May 2022 |
|  | BA.2 | 31 | 25 Mar 2021 | XBB.1.5 | 42 | 12 Jun 2022 |
|  | BA.2.12.1 | 33 | 28 Sep 2021 | BM.4.1.1 | 39 | 20 Jul 2022 |
|  | BA.2.65 | 31 | 11 Oct 2021 | XBC.1 | 39 | 6 Sep 2022 |
|  | BA.1.1.15 | 37 | 27 Nov 2021 | CH.1.1.1 | 42 | 15 Oct 2022 |
|  | BA.5 | 34 | 9 Dec 2021 | XBB.1.16 | 43 | 4 Jan 2023 |
|  | BA.4.1 | 35 | 14 Dec 2021 | EG.1 | 43 | 16 Jan 2023 |
|  | BQ.1.1 | 37 | 20 Dec 2021 | HV.1 | 46 | 29 Jan 2023 |
|  | BA.2.75 | 30 | 31 Dec 2021 | HK.3 | 45 | 29 Jan 2023 |
|  | BF.5 | 35 | 8 Jan 2022 | EG.5.1 | 44 | 31 Jan 2023 |
|  | BF.7 | 35 | 24 Jan 2022 | DV.7.1 | 45 | 29 May 2023 |
| P-lineage | JN.1 | 60 | 13 Jan 2023 | KP.3 | 63 | 7 Jan 2024 |
|  | BA.2.86.1 | 59 | 17 Jan 2023 | LB.1 | 64 | 15 Jan 2024 |
|  | BA.2.86 | 58 | 11 Mar 2023 | KP.1 | 63 | 1 Feb 2024 |
|  | JN.2 | 59 | 22 Jun 2023 | KS.1 | 58 | 15 Feb 2024 |
|  | JN.1.7 | 62 | 25 Sep 2023 | KP.1.1.3 | 65 | 23 Feb 2024 |
|  | JN.1.18 | 57 | 11 Dec 2023 | XDV.1 | 56 | 26 Feb 2024 |
|  | JN.1.11.1 | 62 | 29 Dec 2023 | LP.1 | 66 | 22 Apr 2024 |
|  | KP.3.1.1 | 64 | 1 Jan 2024 | XED | 64 | 19 Jun 2024 |
|  | KP.2 | 59 | 2 Jan 2024 | XEC | 65 | 28 Jun 2024 |
|  | JN.1.37 | 61 | 3 Jan 2024 | LF.7 | 67 | 26 Aug 2024 |
|  | XEB | 61 | 3 Jan 2024 | LF.7.1.2 | 68 | 8 Sep 2024 |
|  | XDQ.1 | 55 | 5 Jan 2024 | LP.8.1 | 68 | 19 Sep 2024 |

* NMS: number of mutated sites. **^‡^** Dates : the worldwide first sample collection date.

**Supplementary Material 2: Phylogenetic tree obtained by cladogenesis algorithm**

**Figure S1** Phylogenetic tree of 25 mutants (total number of mutated sites =104) in 4-letter representation


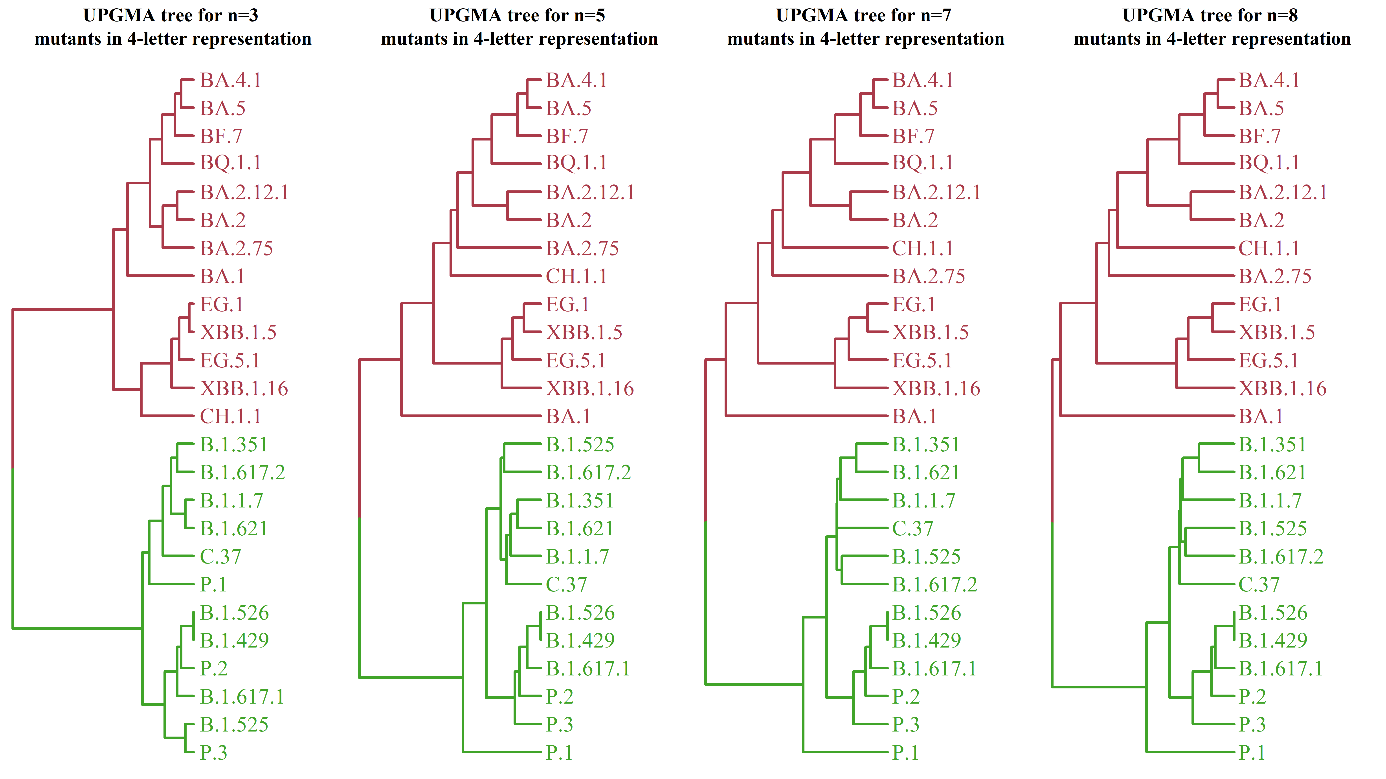


**Figure S2** Phylogenetic tree of 25 mutants (total number of mutated sites =104) in 2-letter representation


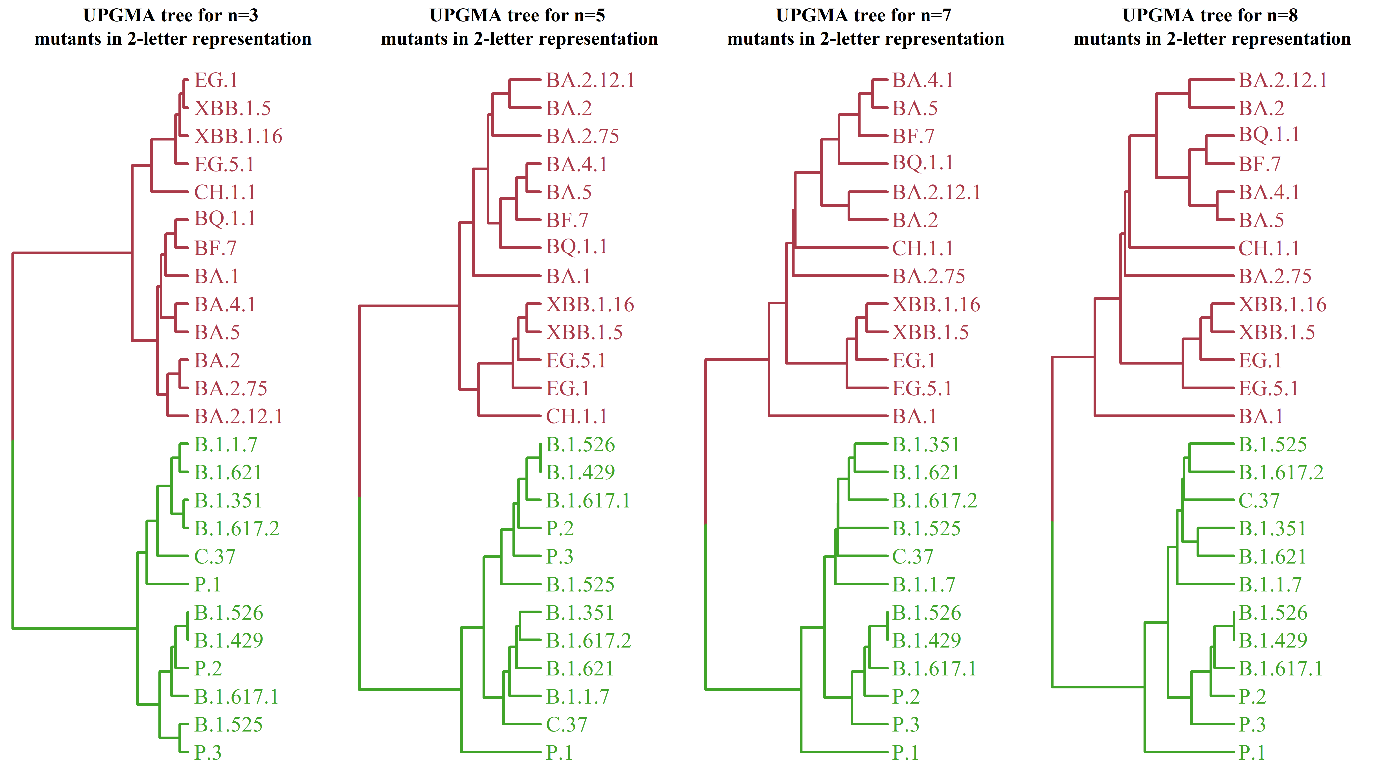


Figures S1and S2 above show the comparison of cladogram structures of phylogenetic trees with different *n* values and letter representations.
